# Single-dose/day versus extended antibiotic prophylaxis for elective urological surgery: a prospective randomized controlled trial

**DOI:** 10.1016/j.eclinm.2026.104303

**Published:** 2026-10-29

**Authors:** Rishi Nayyar, Rajath S. Shetty, Anurag Kushwaha, Sarita Mahapatra, Sridhar Panaiyadiyan, Prashant Singh, Siddharth Jain, Manoj Kumar, Sanjay Kumar, Brusabhanu Nayak, Amlesh Seth

**Affiliations:** a Department of Urology, All India Institute of Medical Sciences (AIIMS), New Delhi, India; b Department of Microbiology, All India Institute of Medical Sciences (AIIMS), New Delhi, India

**Keywords:** Antibiotics, Urinary tract infection, Peri-operative prophylaxis, Urological surgery, Antimicrobial stewardship, Bacteriuria, Endourology

## Abstract

**Background:**

Excess and prolonged antibiotic use for perioperative prophylaxis is a very common clinical practice. This overuse is largely driven by ingrained protocols rooted in fear of postoperative infection, despite limited evidence supporting extended prophylaxis. The objective was to evaluate whether extending antibiotic prophylaxis beyond the immediate perioperative period provides additional protection against postoperative symptomatic urinary tract infection (UTI) in patients undergoing elective urological surgery.

**Methods:**

This open-label, randomized controlled trial included patients scheduled for elective urological surgery from March 2024 to September 2025. The patients were randomized 1:1 to either single-dose or extended perioperative prophylaxis. The primary endpoint was symptomatic urinary tract infection (sUTI) within 30 days of surgery. Secondary endpoints included one-week sUTI rate, asymptomatic bacteriuria at 7 and 30 days, 30-day hospital readmission, antibiotic escalation, length of stay and subgroup analyses by age, co-morbidity, surgical approach, and procedure type. The trial was prospectively registered with the Clinical Trials Registry of India (CTRI/2024/03/063953).

**Findings:**

A total of 334 patients completed follow-up and were included in the analysis (single-dose group: n = 169; extended-prophylaxis group: n = 165). The 30-day sUTI rate was 7.1% (12/169) in single versus 5.5% (9/165) in extended prophylaxis groups (Absolute risk difference 1.6%, 95% CI: −3.6% to 6.8%; p = 0.536). All secondary outcome parameters were comparable between the groups with no statistically significant difference. No patient in either group developed septic shock or infection-related mortality.

**Interpretation:**

As a test of difference, without implying equivalence or non-inferiority, there was no statistically significant difference in postoperative symptomatic UTI incidence between single-dose and extended antibiotic prophylaxis for elective urological surgery.

**Funding:**

None.

**Research in context**

**Evidence before this study**

Excess and prolonged pre- or post-operative antibiotic use remains very common in uro-surgical practice. This is because the existing evidence support in form of randomized trials for single-dose prophylaxis is very limited and largely based on studies that evaluated individual procedures or specific patient populations, and concerns remain about 30-day UTI rates or sepsis. To the best of our knowledge, there is currently no randomized trial that includes broad spectrum of urological surgeries and diseases to compare efficacy of single-dose versus extended antibiotic prophylaxis.

**Added value of this study**

In this prospective randomized controlled trial involving 334 patients undergoing elective urological surgery, single-dose prophylaxis was associated with similar rates of 30-day postoperative symptomatic urinary tract infection, sepsis, asymptomatic bacteriuria, hospital stay, antibiotic escalation, and readmission compared with extended prophylaxis.

**Implications of all the available evidence**

These findings support the use of single-dose perioperative antibiotic prophylaxis as a standard policy for most elective urological surgeries. Further, equivalence studies are advisable to confirm these findings. As extended antibiotic use goes against the principle of antimicrobial stewardship, a uniform single-dose antibiotic policy may also help improve adoption of stewardship programs in institutions rather than an individual case-based bargain approach.

## Introduction

Antibiotic prophylaxis is a cornerstone of perioperative care in urological surgery, aimed at reducing postoperative infectious complications while minimizing unnecessary antimicrobial exposure. Compared with other surgical specialties, urology presents unique challenges because of routine instrumentation of the urinary tract, exposure to urine containing uro-pathogens, use of irrigation under pressure during endourological procedures, and frequent placement of foreign bodies such as ureteral stents and catheters. As a result, postoperative urinary tract infection (UTI) remains a clinically relevant concern across a wide spectrum of urological procedures.^1^

Current international guidelines consistently recommend single-dose or short-duration perioperative antibiotic prophylaxis for most elective urological surgeries when preoperative urine is sterile.^2^ Despite available evidence the assertiveness as well as acceptability of these recommendations is limited and the actual clinical practice is hugely aberrant.^2^, ^3^ This may be due to skepticism about applicability of these recommendations owing to variations in several confounding factors including but not limited to aseptic practices, patient socio-economic profile, disease profile, weather-profile, bacterial bio-gram etc. Concerns regarding postoperative fever, gram-negative bacteremia, sepsis, and their medico-legal implications frequently drive prolonged antibiotic prescriptions, particularly after endourological stone procedures and complex surgeries. Increasing evidence, however, suggests that prolonged prophylaxis does not reduce postoperative infection rates and may contribute to antimicrobial resistance, adverse drug effects, fungal infections and increased healthcare costs. More and more studies are required to generate high-quality evidence with respect to risk-benefit in different practice and regional settings.

Most prior studies have focused on individual procedures and/or have been conducted in resource-rich environments and often relied on surrogate outcomes such as postoperative fever. Randomized data evaluating clinically meaningful outcomes, such as 30-day symptomatic UTI, across a broad range of urological procedures remain limited. The present study was therefore designed to compare single-dose perioperative antibiotic prophylaxis with extended postoperative prophylaxis using clinically relevant endpoints in a real-world hospital setting.

## Methods

### Study design and setting

This was a prospective randomized open-label study conducted at a tertiary referral center between March 2024 and September 2025. The study was approved by the Institutional Ethics Committee (A00709/2024) and prospectively registered with the Clinical Trials Registry of the country (CTRI/2024/03/063953) and has been reported in compliance with CONSORT guidelines.

### Ethics

Written informed consent was obtained from all participants aged 18 years or older. For participants younger than 18 years, written informed consent was obtained from a parent or legally acceptable representative, and assent was obtained separately from the child in accordance with the Indian Council of Medical Research national ethical guidelines (2017).

### Study population

Patients aged 5–75 years undergoing elective urological surgery were screened for eligibility, indicative of majority case-load in our unit. Procedures included endoscopic, minimally invasive, and open urological surgeries. Patients with active clinical infection requiring antibiotics before or at the time of surgery were excluded. Data on race and ethnicity were not collected.

Exclusion criteria included refusal of consent, surgeries involving entry into the gastrointestinal tract, reconstructive procedures, artificial implant placement, WHO class III or IV wounds, bilateral surgery, immunocompromised state, Charlson Comorbidity Index ≥5, pregnancy, high risk of infective endocarditis, significant intraoperative blood loss (>1 L), intraoperative deviation requiring unplanned intervention, and neuro-urological patients requiring chronic or intermittent catheterization.

### Randomization and allocation

Eligible patients were randomized in a 1:1 ratio using a computer-generated sequence to receive either single-dose perioperative antibiotic prophylaxis or extended postoperative prophylaxis. Randomization was not stratified by procedure type or surgical approach. Allocation was concealed using sequentially numbered sealed envelopes of the computer-generated random sequence, with allocation revealed at the time of enrollment prior to surgery.

### Antibiotic protocol

Patients with sterile preoperative urine cultures received intravenous ceftriaxone 1 gm as prophylaxis policy. Patients with positive culture received the antibiotic based on culture sensitivity using the lowest order of intravenous antibiotic. This meant use of any one antibiotic from the second-generation cephalosporin, fluoroquinolone, penicillin, aminoglycoside, penicillin + beta-lactam inhibitor, third or fourth-generation cephalosporin, or the carbapenem group antibiotic in that order. Dual antibiotic drugs were not allowed for prophylaxis.

The single-dose group received the antibiotic at induction of anesthesia, with or without an additional dose on postoperative day 0. The extended-prophylaxis group received intravenous antibiotic postoperatively followed by oral cefixime to complete a total of 12 doses. Apart from antibiotic duration, the perioperative hospital care [including surgical technique, sterile precautions, wound management, catheter handling, step-down care etc] was standardized and similar for both groups. Antibiotic escalation and/or prolongation were permitted in both the groups for the treatment of symptomatic postoperative UTI or systemic inflammatory response syndrome (SIRS) as clinically indicated and at the discretion of the operating surgeon.

### Outcome measures

The primary outcome was symptomatic UTI (sUTI) within 30 days postoperatively, defined by new urinary symptoms (dysuria, urinary frequency, urgency, suprapubic discomfort, flank pain, or testicular pain and swelling) or fever (>38°C) suggestive of infection. Microbiological confirmation was not mandatory reflecting real-world postoperative practice. Any systemic inflammatory response syndrome, infection related ICU admission was also recorded.

Pre-specified secondary outcomes included: (i) The incidence of sUTI within the first postoperative week; (ii) The rate of asymptomatic bacteriuria at one week and at 30 days; (iii) 30-day readmission/consultation rate related to infectious complications; (iv) The need for antibiotic escalation/prolongation during the study period; (v) Length of hospital stay; and (vi) Subgroup comparative analysis of UTI rates stratified by age group, co-morbidity, surgical approach, and procedure type.

### Microbiological assessment

Urine cultures were obtained from all participants at three defined time points: preoperatively, at one week, and at 30 days postoperatively. Additional cultures were obtained at any point in time where clinical suspicion of infection arose. Asymptomatic bacteriuria was defined as a colony count of ≥10^5^ colony-forming units per milliliter (CFU/mL) in a midstream urine specimen in the absence of symptoms attributable to UTI.

### Follow-up

All participants were assessed clinically during their inpatient stay for any sUTI or adverse drug reactions and reviewed again at a scheduled postoperative outpatient visit and at one month following surgery. Follow-up included structured symptom assessment, temperature recording, physical examination, and laboratory investigation as clinically indicated.

### Sample size calculation

There are no similar prior studies that report on incidence of sUTI in a population covering broad range of urological surgeries as a whole. In a study by Chew et al. postoperative UTI rates of 4.7% and 15.3% were reported for single-dose and extended antibiotic prophylaxis respectively, following ureteroscopy.^4^ Assuming a comparable inter-group difference of approximately 10 percentage points in postoperative UTI incidence, a total sample of 334 evaluable patients was determined considering 90% statistical power at a two-sided significance level of 5%. Sample size calculations were performed using G∗Power 3.1.9.4 software. To accommodate anticipated dropout and protocol deviations, 359 patients were enrolled.

### Statistical analysis

All data were entered and managed using Microsoft Excel and analyzed with SPSS version 26·0. Descriptive statistics were employed to characterize baseline demographic and clinical variables. Categorical outcomes, including the primary endpoint of postoperative symptomatic UTI, were compared between groups using the chi-square (χ^2^) test. Where expected cell frequencies fell below five, Fisher's exact test was applied. Continuous variables were tested for normality using the Shapiro–Wilk test; non-normally distributed variables, such as hospital length of stay, were compared using the Mann–Whitney U test. Both per-protocol and intention-to-treat analysis were performed. The absolute risk difference for the primary outcome was reported with its 95% CI, calculated using the Wald method. No non-inferiority or equivalence margin had been prespecified, so the primary comparison was interpreted as a test of difference, with emphasis on the precision of the estimate rather than on the p value alone. A two-sided p-value of less than 0.05 was considered statistically significant for all comparisons. Subgroup analyses were exploratory.

### Role of the funding source

There was no funding source for this study.

## Results

### Study population

A total of 359 patients were assessed for eligibility. Of these, 181 patients were allocated to the single-dose prophylaxis group and 178 to the extended prophylaxis group. During the study period, no patients were lost to follow-up. A total of 25 patients were excluded from the final analysis due to protocol deviations, 12 from the single-dose group and 13 from the extended group (Fig. 1). A per-protocol analysis was done because the deviations from the protocol were primarily related to requirement of an additional procedure during the surgery itself or in the postoperative period. These additional procedures necessitated additional antibiotic doses and constituted exclusion criteria. There were only two deviations from protocol on account of antibiotic drug or its dosing in both groups.

**Fig. 1:**
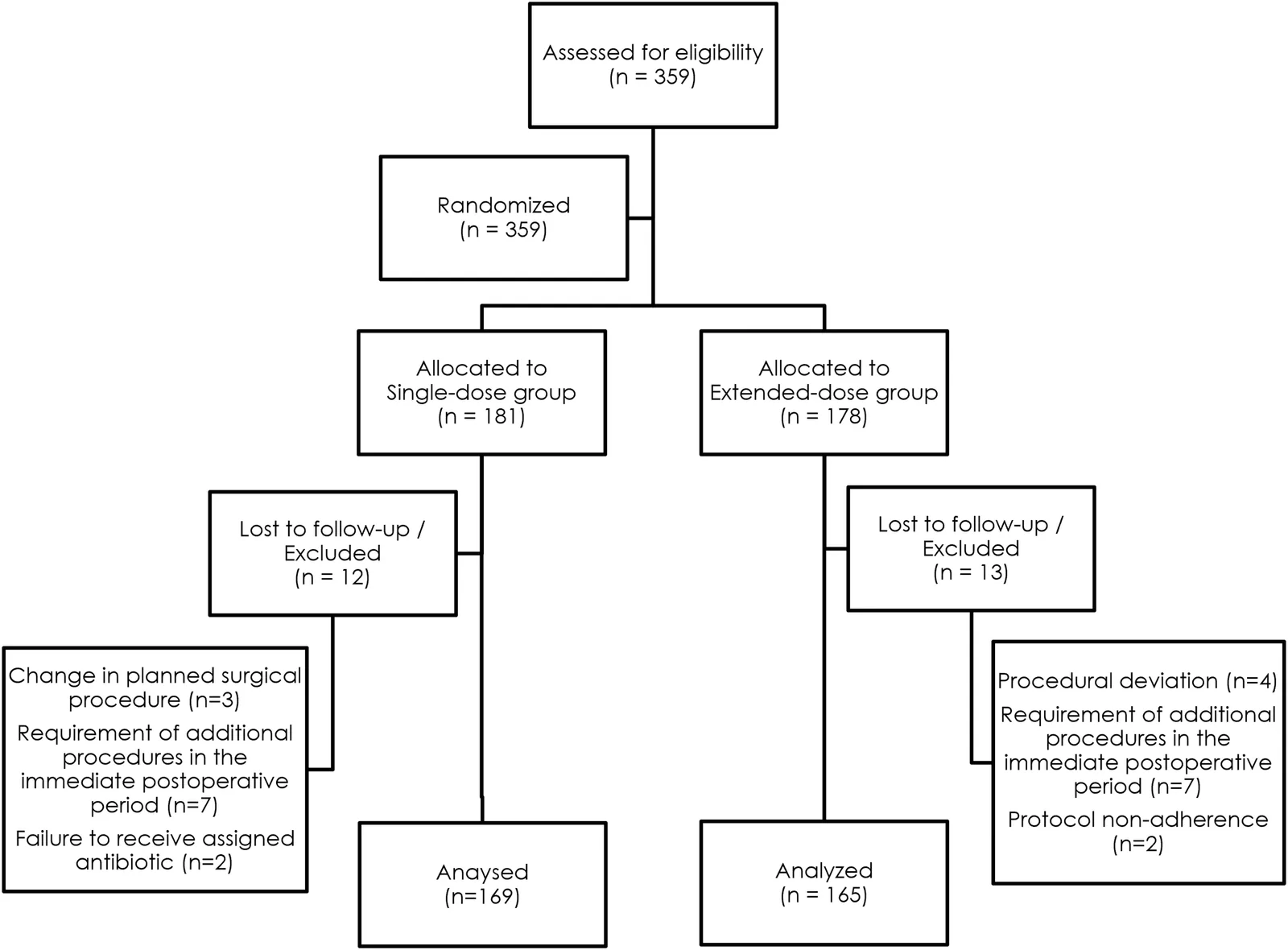
Patient flow summary (CONSORT format).

334 patients were included in the final analysis: 169 in the single-dose group and 165 patients in the extended prophylaxis group. 142 (84.0%) in the single-dose arm received one dose only. In the extended prophylaxis group, the mean number of intravenous antibiotic doses administered was 4.22, with a median of 5 doses (IQR 3–5) prior to conversion to oral therapy. All patients ultimately received a total of 12 antibiotic doses.

### Baseline characteristics

Baseline characteristics were mostly balanced between the two groups (Table 1) except for distribution of PCNL and TURP as procedure type. Overall comorbidity burden was low. The prevalence of hypertension (16.0% versus 18.2%), diabetes mellitus (10.1% versus 12.1%), and chronic obstructive pulmonary disease (1.2% versus 3.0%) did not differ significantly between groups (all p > 0.05). Preoperative urine culture positivity was low and comparable in both cohorts (3.0% versus 1.8%; p > 0.05). Endoscopic procedures were the dominant procedure type in both groups. Intraoperative blood transfusion rates were comparable between groups (3.6% versus 5.5%; p > 0.05), indicating equivalent operative complexity.

**Table 1:** Baseline demographic and clinical characteristics.

Parameter	Single-dose prophylaxis (n = 169)	Extended prophylaxis (n = 165)	p-value
Age, mean ± SD (years)	48.2 ± 17.16	48.22 ± 17.23	NS
Sex: Male/Female, n	127/41	123/40	NS
Charlson Comorbidity Index, mean	0.12	0.15	NS
Hypertension, n (%)	27 (16.0)	30 (18.2)	NS
Diabetes mellitus, n (%)	17 (10.1)	20 (12.1)	NS
COPD, n (%)	2 (1.2)	5 (3.0)	NS
Pre-op urine culture positive, n (%)	5 (3.0)	3 (1.8)	NS
Surgical approach: Endo/MIS/Open, n	109/47/13	116/36/13	NS
Blood transfusion, n (%)	6 (3.6%)	9 (5.5%)	NS
Surgical procedure distribution, n (%)			
TURBT	46 (27.2)	43 (26.1%)	0.90
TURP	21 (12.4)	8 (4.8)	0.018
URSL	18 (10.7)	12 (7.3)	0.34
RIRS	7 (4.1)	13 (7.9)	0.17
PCNL	12 (7.1)	32 (19.4)	0.001
Nephrectomy (open + MIS)	22 (13.0)	20 (12.1)	0.87
Partial nephrectomy	10 (5.9)	14 (8.5)	0.40
Pyeloplasty	7 (4.1)	4 (2.4)	0.54
Radical prostatectomy	6 (3.6)	4 (2.4)	0.75
Nephroureterectomy	1 (0.6)	2 (1.2)	0.62
Miscellaneous	19 (11.2)	13 (7.8)	0.60

MIS = minimally invasive surgery; CCI = Charlson Comorbidity Index; COPD = chronic obstructive pulmonary disease; TURBT = transurethral resection of bladder tumor; TURP = transurethral resection of prostate; URSL = ureterorenoscopic lithotripsy; RIRS = retrograde intrarenal surgery; PCNL = percutaneous nephrolithotomy. NS = not significant (p > 0.05). All p-values derived from Fisher's exact test or Mann–Whitney U test as appropriate.

### Primary outcome

In the per-protocol analysis, sUTI at 30 days occurred in 12 of 169 patients (7.1%) in the single-dose group and 9 of 165 patients (5.5%) in the extended prophylaxis group, representing a statistically non-significant absolute risk difference of 1.6% (95% CI: −3.6% to 6.8%) and an overall rate of 6.2% across the entire cohort (Table 2). This difference was not statistically significant (χ^2^ test, p = 0.536). Barring two deviations in either group related to antibiotic drug or its dose, rest all deviations from the protocol were primarily related to requirement of an additional procedure during or after the planned surgery (Fig. 1). Considering intention-to-treat analysis, 30 days sUTI occurred in 14 (7.7%) of 181 patients in the single-dose group and 11 (6.2%) of 178 patients in the extended prophylaxis group. The absolute risk difference was 1.6 percentage points (95% CI: −3.7 to 6.8) and the difference remained not statistically significant (χ^2^ test, p = 0.563).

**Table 2:** Incidence of 30-day postoperative symptomatic UTI (primary outcome).

Group	Patients (N)	UTI cases (n)	UTI rate (%)	p-value	Risk difference and Confidence interval
Single-dose prophylaxis	169	12	7.1%	0.536	1.6% (95% CI: −3.6% to 6.8%)
Extended prophylaxis	165	9	5.5%		
Total	334	21	6.2%	χ^2^ test	

To account for the imbalance in distribution of procedure types (PCNL and TURP) between the groups, the primary outcome was re-analyzed by logistic regression with procedure category as a covariate. After this adjustment, the odds of 30-day sUTI with single-dose prophylaxis relative to extended prophylaxis were 1.71 (95% CI: 0.68–4.33; p = 0.26). Procedure category itself was strongly associated with the outcome, with upper-tract stone endoscopy carrying the highest event rate (12.8%, 12/94) and lower-tract endoscopy the lowest (1.6%, 2/129). The direction of the treatment estimate was unchanged by adjustment and remained statistically non-significant, but the confidence interval is wide and does not exclude a clinically relevant difference in either direction.

Among the 21 patients who developed sUTI, clinical presentation spanned a range of severity. In the single-dose group, 11 patients developed UTI during their index hospital admission, of which 9 had post-operative fever presumed as pyelonephritis, 2 developed sepsis without progression to septic shock, and 1 experienced postoperative orchitis requiring culture-based antibiotics. One additional patient was readmitted following discharge with fever and systemic features requiring intravenous antibiotics and escalation to piperacillin and tazobactam. In the extended prophylaxis group, 8 patients developed infection prior to discharge (6 with postoperative fever, 2 with sepsis without shock), and 1 patient was readmitted after discharge with culture-positive UTI and systemic features requiring intravenous antibiotics and escalation to piperacillin and tazobactam. No patient in either group required vasopressor support or intensive care unit admission or developed septic shock, organ dysfunction, or infection-related mortality during the 30-day follow-up period.

### Secondary outcomes

#### One-week sUTI rate

All early UTI events occurred prior to hospital discharge in both groups. The difference in 1-week sUTI was not statistically significant (χ^2^ test, p = 0.52; absolute risk difference 1.7%, 95% CI: −3.4% to 6.8%) between the two groups.

#### Thirty-day readmission rate

Readmission attributable to postoperative infectious complications occurred in one patient (0.6%) in each group within 30 days of surgery (p = 1.00, Fisher's exact test). Both readmitted patients responded to intravenous antibiotic therapy and were discharged without further sequelae or ancillary procedures.

#### Asymptomatic bacteriuria

Asymptomatic bacteriuria rates were comparable between groups at both 1 week and 30 days assessment time points (Table 3) with no statistically significant difference.

**Table 3:** Asymptomatic bacteriuria at one week and thirty days.

Time point	Single-dose, n (%)	Extended, n (%)	p-value
1 week postoperative	23 (13.6%)	20 (12.1%)	0.69
30 days postoperative	11 (6.5%)	10 (6.1%)	0.88

#### Subgroup analysis

Subgroup analysis by Age [≤10, 11–69, or ≥70 year]; Surgical approach [Open, Endoscopic, or, Minimally invasive Laparoscopic or Robotic]; Procedure type [TURBT, TURP, URSL, RIRS, PCNL, nephrectomy, partial nephrectomy, pyeloplasty, radical prostatectomy, etc]; Co-morbidities [Diabetes mellitus, Hypertension, or hypothyroidism]; and, Presence of preoperative urinary drainage [Double-J ureteric stent, Per-urethral catheter, Percutaneous nephrostomy tube, or None]; all yielded no statistically significant difference (Supplementary File).

#### Length of hospital stay

The median duration of hospital stay was 2 [Interquartile range: 2–3] days in the single-dose group and 2 [Interquartile range: 2–4] days in the extended prophylaxis group. With no statistically significant difference between the groups (Mann–Whitney U test) (p = 0.27).

#### Antibiotic escalation

Escalation to a broader-spectrum antibiotic regimen was required in 5 patients (3.0%) in the single-dose group and in 4 patients (2.4%) in the extended prophylaxis group (Fisher's exact test, p = 1.00).

### Detailed microbiological and clinical characteristics of symptomatic UTI cases

A structured summary of the clinical and microbiological profile of all 21 symptomatic UTI cases is provided in Table 4. All four patients of RIRS that developed UTI had a preoperative DJ stent in situ for 14 days or more, and the patient of TURBT in the single-dose group had a preoperative PCN in situ. Only four out of 21 presenting with sUTI were found to have positive culture—*E. coli* in three and *Pseudomonas aeruginosa* in one.

**Table 4:** Microbiological and clinical characteristics of symptomatic UTI cases.

Parameter	Single-dose (n = 12)	Extended (n = 9)	Total (n = 21)
Pre-op culture			
Positive	3	0	3
Negative	9	9	18
Post-op culture			
Positive	3	1	4
Negative	9	8	17
Antibiotic escalation			
Required	5	4	9
Not required	7	5	12
Imaging/procedure-confirmed complication	3	3	6
Readmission within 30 days	1	1	2
Procedure type, n			
PCNL	3	3	6
RIRS	3	1	4
Partial nephrectomy	2	0	2
URSL	1	2	3
TURBT	1	0	1
Open nephrectomy	1	0	1
Laparoscopic nephrectomy	0	3	3
Cystolithotripsy	1	0	1
Diabetes mellitus	4	3	7

Associated confirmed procedure-related complications were seen in three patients in each group. In the single-dose group these comprised a PCNL-associated pleural effusion requiring intercostal drainage, a stitch-line infection following open nephrectomy, and a delayed perinephric collection following RIRS. In the extended-prophylaxis group, these included a PCNL-associated hemothorax requiring intercostal drainage and blood transfusion, and one patient each with residual stones following PCNL and RIRS respectively.

### Adverse events

No adverse drug reactions directly attributable to the study antibiotics were recorded in either group and no patient was found to have drug sensitivity before antibiotic administration.

## Discussion

The use of peri-operative antibiotic prophylaxis is highly variable at all levels including surgical procedure type, approach type, surgeon choice, hospital type, co-morbidity type, etc. Coupling this variable practice with variations in antibiotic type, augmented regimens, duration and route of administration leads to extremely non-congruent practice of peri-operative antibiotic prophylaxis.^3^ Thus, there is need to generate more evidence covering all major urological procedures and patient profiles across geographical healthcare settings to bring greater uniformity in urological perioperative antibiotic usage.

Our study results showed that as a test of difference, 30-day sUTI was no different between single dose/day and extended antibiotic prophylaxis for urological surgeries, demonstrating no added clinical benefit from additional antibiotic doses. The one-week UTI rate, asymptomatic bacteriuria at one week and 30 days, 30-day hospital readmission, need for antibiotic escalation, and length of hospital stay were not statistically different. This evidence was generated in the context of different types of elective urological procedures endoscopic, laparoscopic, and open at a high-volume tertiary center in a tropical weather country with relative resource-limited setting. Practice of over-prescribing antibiotics is a concerning worldwide phenomenon that demands urgent attention and engagement from the surgical teams with more data generation and enforcement of evidence-based practices to reduce excess antibiotic usage given the context of rising antimicrobial resistance, adverse effects, drug-drug interactions, associated cost and fungal infections related to antibiotics overuse. 5, 6, 7, 8

Our results also indicate that urological surgeries likely perform similarly to other Class 1 or 2 WHO surgeries where a single-dose prophylaxis is valid, safe and adequate.^9^ During endourological surgeries bacteria from the urine, the stone surface, the urine diversion tube, or the mucosal wall can enter the bloodstream through small pyelovenous and pyelolymphatic back-channel pathways predisposing to bacteremia. However, our data reinforce that even during endourological surgery, the critical window is the procedure itself, not the days that follow. A single dose/day prophylaxis can adequately provide prophylaxis for this critical period with no added advantage from continued dosing. This principle was established by Classen et al. in 1992^10^ and has since been built into every major surgical antibiotic guideline that followed.

Our findings are broadly consistent with the published literature across all major procedure categories. A single intraoperative antibiotic dose has been found to be sufficient to prevent postoperative UTI after ureteroscopy in large studies and metaanalysis.^4^, ^11^ Studies have looked specifically at perceived high-risk patient groups with pre-existing ureteric stents, recurrent UTI histories, or prior procedural failures and have found no reduction in infectious complications with prolonged prophylaxis.^12^, ^13^ For percutaneous nephrolithotomy, the evidence is similarly clear. Trials have shown no benefit from extended preoperative courses in culture-negative PCNL patients.^14^, ^15^ Jung et al.'s meta-analysis of 15 PCNL trials found that continuing antibiotics after the procedure did not reduce infection and was associated with more postoperative fever, not less.^16^ For transurethral procedures, laparoscopic nephrectomy, and robotic prostatectomy, single-dose prophylaxis has similarly been shown to be adequate in low-to-moderate risk patients in published studies.^17^, ^18^ One finding from the broader literature that deserves particular emphasis is the observation by Calvert et al. that extended postoperative antibiotic prophylaxis following major urological cancer surgery was independently associated with higher rates of *Clostridium* *difficile* infection without any reduction in surgical site or urinary tract infections.^19^ This is a real and serious harm delivered by the practice of extended prophylaxis, and it needs to be weighed more heavily in institutional prescribing decisions than it currently does. Cai et al. demonstrated in a multicentre study that following guideline-recommended short prophylaxis was associated with equivalent or better perioperative outcomes than otherwise, confirming that this approach is clinically safe in routine practice.^20^

While it is fathomable that urinary diversion tubes, positive urine culture and stone-surgeries are risk factors for postoperative UTI, excess antibiotic use may not be an automatic mitigating strategy, unless proved otherwise. We did not find any difference of sUTI rates in any of such subgroups of patients. The following points need to be understood in this regard. Post-op UTI is rarely caused by same organism as grown on pre-op mid-stream urine culture.^21^ Pre-op or post-op prolonged antibiotic does not selectively target the organism grown on pre-op culture but disturbs the entire biological milieu of the body.^22^ Excess antibiotic usage as prophylaxis cannot eliminate the bacteria from human body. Rather, it selecting out more resistant bacteria which ultimately may become a risk-factor for more severe forms of UTIs.^23^

The question of asymptomatic bacteriuria deserves separate attention because it is frequently used often implicitly to justify continued and prolonged antibiotic therapy before or after endourological procedures. Even in our trial we found it difficult to recruit cases with preoperative asymptomatic bacteriuria, despite no restriction to this effect in the study protocol and some recent large prospective studies failing to identify asymptomatic bacteriuria as a predisposing factor for post-operative UTI.^24^, ^25^ Most of earlier randomized trials have failed to recruit cases with asymptomatic bacteriuria given the lack of consensus guidelines on this specific group of patients.

Another lacuna of most available studies dealing with perioperative prophylaxis in urological surgeries is that they use fever as the primary end-point, and project it as a surrogate for the more-important end-point of septicemic shock or need for ICU care. Some have even used reduced post-operative bacteriuria rates as a surrogate marker to justify prolonged antibiotic use. These appear to be important fallacies because most fevers/UTI, if they occur, can be easily cured with subsequent continuation or restart of antibiotics without any residual untoward effect. Whereas, septicemic shock or need for ICU care are more important parameters, but occur only in 1–2% cases. Such use of fever/clinical UTI or bacteriuria rates as primary end-point induces a false sense of essentiality for continuing antibiotic prophylaxis in perioperative period. Even with single-day/dose protocols for prophylaxis, no patient is denied continuity of care. If and when local or systemic UTI occurs, antibiotic(s) can be continued, restarted or escalated with equivalent efficacy as found in our study as well. In our trial, bacteriuria rates at one week (13.6% versus 12.1%, p = 0.69) and 30 days (6.5% versus 6.1%, p = 0.88) were similar between groups despite the extended prophylaxis arm receiving more than five times as many antibiotic doses. Extended courses suppress bacteriuria transiently but once stopped, re-colonization occurs at roughly the same rate regardless of prior antibiotic duration.^12^, ^26^ Antibiotic escalation was required in only 5 patients (3.0%) in the single-dose group and 4 patients (2.4%) in the extended prophylaxis group a difference that was not statistically significant (p = 1.00), in our study, which is clinically reassuring with no septic shock, ICU admission, residual sequalae or mortality from infection during the 30-day follow-up.

Often post-op fever/UTI is associated with a surgical complication like hematuria with clot retention, hydropneumothorax or chest infection which are fever/infection driven by the nature and complexity of the procedures performed and its complications. Some of these complications were seen in our study as well and were part of the subset where antibiotic escalation was done in either of the groups. These events would likely have occurred regardless of the prophylaxis strategy, because they reflect intraoperative events that postoperative antibiotics cannot prevent and where clinical course was determined by other factors.

The broader relevance of these findings in the healthcare context of our country and in general per se cannot be overstated, particularly in the contemporary context of already advanced antimicrobial resistance. Fluoroquinolone resistance in urinary *E. coli* exceeds 60% at many Indian tertiary centers, and resistance to cephalosporins the antibiotic class used for prophylaxis in this trial is rising at a rate that threatens the long-term utility of these agents. Gandra et al. have described the resulting resistance landscape as a compounding crisis shaped directly by years of permissive antibiotic use.^27^, ^28^ A 2025 multicentric survey documented that surgical specialties including urology, are among the leading contributors to antibiotic overprescribing burden at Indian tertiary hospitals.^29^ Against this background, our earlier national audit showing that single-dose prophylaxis is used in fewer than one in five Indian urological procedures is particularly striking.^3^

Overall, this trial adds to the published literature in a specific way that prior evidence has not covered. Most existing studies are restricted to a single procedure type and were conducted in Western settings with different patient populations, pathogen profiles, and healthcare environments. This trial enrolled patients undergoing many different procedure types including endoscopic, minimally invasive, or open procedures at an Indian academic center and followed all of them for 30 days The overall postoperative infection rate observed in the present study (6.2%) is comparable to the rates reported in published literature evaluating postoperative urinary tract infections following urological and endourological procedures.^4^, ^11^ Our demonstration that single-dose prophylaxis is equivalent to extended prophylaxis across this full procedural range strengthens the case for a single uniform institutional policy rather than requiring individual negotiations for each procedure type.

The main limitations of this trial are an open-label design meaning that clinicians and patients knew which group each patient was in, which may have influenced how symptoms were reported and assessed. However, this effect was somewhat mitigated by a mandatory 1-week and 30-day follow-up strategy and pre-defined definitions of sUTI. Symptomatic UTI was defined clinically without requiring a positive urine culture in every case reflecting the real-world diagnostic practice but introduces some subjectivity. The documented culture-positivity in only four out of 21 sUTI cases ends up only reinforcing confidence in the definition used for the primary outcome. Also, even though the sample-size calculation was based on an anticipated inter-group difference in postoperative sUTI incidence, a formal non-inferiority or equivalence margin was not prospectively prespecified. Therefore, the absence of a statistically significant difference between the two groups should not be interpreted as demonstrating statistical equivalence or non-inferiority. The upper limit of the 95% CI for the intention-to-treat risk difference (6.8 percentage points) means that a clinically important increase in sUTI with single-dose prophylaxis cannot be absolutely excluded. The overall event rate was also lower than anticipated, which reduced the precision of the estimate. Further, the procedure-specific subgroups were too small to draw reliable procedure-level conclusions and not the intent of the study. The septicemia events were very low for meaningful comparisons, though it also indicates that fever/clinical UTI by themselves may not be good end-points for such studies. Additionally, the study was conducted at a single center, which may limit the generalizability of the findings and more such studies should be done to reaffirm the findings across all practice regions and types. It is only through such studies that one may expect stricter compliance with guideline recommendations. Also, this study could not recruit many cases with preoperative asymptomatic bacteriuria, which remains an important area of management in urological surgeries.

Overall, this study strengthens confidence in the use of single dose/day antibiotics across a broad range of urological procedures as a whole and may be used for guideline recommendations accordingly.

A single dose/day perioperative antibiotic strategy is not associated with a statistically significant difference in postoperative 30-day sUTI from an extended multi-dose postoperative regimen for elective urological surgery. These findings do not show a benefit from routine prolongation of perioperative antibiotic prophylaxis in the population studied.

## Contributors

All authors had full access to all the data in the study and accepted responsibility for the decision to submit for publication.

RN: Conceptualization, data curation, investigation, methodology, project administration, supervision, validation, visualization, and writing—review & editing, accessed and verified the underlying data.

RSS: Data curation, formal analysis, investigation, methodology, software, visualization, validation, writing—original draft, accessed and verified the underlying data.

AK: Data curation, investigation, software, visualization, writing—original draft, accessed and verified the underlying data.

SM: Data curation, investigation, methodology, project administration, resources, and writing—review & editing.

SP: Data curation, investigation, methodology, writing—review & editing.

PS: Data curation, investigation, methodology, writing—review & editing.

SJ: Data curation, investigation, methodology, writing—review & editing.

MK: Data curation, investigation, methodology, writing—review & editing.

SK: Data curation, investigation, methodology, writing—review & editing.

BN: Data curation, investigation, methodology, writing—review & editing.

AS: Data curation, investigation, methodology, writing—review & editing.

## Data sharing statement

Deidentified study data are available from the corresponding author upon reasonable request.

## Declaration of interests

All authors declare no competing interests.

## Appendix A Supplementary data

Supplementary Tables

Protocol
