# Supplementary material for: Single-dose/day versus extended antibiotic prophylaxis for elective urological surgery: a prospective randomized controlled trial
Source: eClinicalMedicine. 2026 Oct 29;101:104303. doi: 10.1016/j.eclinm.2026.104303 (PMC13742308; doi:10.1016/j.eclinm.2026.104303)
Supplement: Supplementary file 1 [file mmc1.docx]

**Supplementary Tables**

**Table S1. Uropathogens and antimicrobial susceptibility profile in pre-operatively urine culture positive cases**

| **Organism** | **Isolates (n)** | **Specimen Type** | **Susceptibility Pattern** |
| --- | --- | --- | --- |
| ***Escherichia coli*** | 4 | Urine (n=4) | Ceftriaxone: 3/4 susceptible; Piperacillin-tazobactam: 4/4; Amikacin: 4/4; Nitrofurantoin: 4/4; Ciprofloxacin: 2/4; Meropenem: 4/4 |
| ***Klebsiella*** | 2 | Urine (n=2) | Piperacillin-tazobactam: 2/2; Amikacin: 2/2; Meropenem: 2/2; Ceftriaxone: 0/2 |
| ***Enterococcus species*** | 1 | Urine (n=1) | Amoxicillin: 1/1; Vancomycin: 1/1; Linezolid: 1/1 |
| ***Pseudomonas aeruginosa*** | 1 | Urine and nephrostomy (paired) | Piperacillin-tazobactam: 1/1; Amikacin: 1/1; Meropenem: 1/1; Ceftriaxone: 0/1 |

**Table S2. Clinical context of Postoperative culture-positive patients that had sUTI**

| **Patient** | **Group** | **Organism** | **Specimen** | **Clinical Context** |
| --- | --- | --- | --- | --- |
| 1 | Single-dose | Escherichia coli | Urine | Postoperative fever; responded to antibiotic escalation |
| 2 | Single-dose | Escherichia coli | Urine | Postoperative fever; required antibiotic escalation |
| 3 | Single-dose | Pseudomonas aeruginosa | Urine + Nephrostomy | Major postoperative haemorrhagic complication; paired cultures positive |
| 4 | Extended | Escherichia coli | Urine | Readmitted with fever; responded to intravenous antibiotics |

**Table S3. Duration of hospital stay**

| **Group** | **Median (days)** | **IQR (days)** | **Range** |
| --- | --- | --- | --- |
| **Single-dose (n=169)** | 2 | 2–3 | 1–8 |
| **Extended (n=165)** | 2 | 2–4 | 1–10 |

**Table S4. Clinical context of post-operative urethral catheter, nephrostomy and double J stent**

| **Drainage Type** | **Single-dose (n=169)** | **Extended (n=165)** | **p-value** |
| --- | --- | --- | --- |
| **Per-urethral catheter** |  |  |  |
| Median (IQR) duration | 2 (1–3) days | 2 (1–3) days | 0·68 |
| Range | 1–14 days | 1–14 days |  |
| **Ureteric stent** |  |  |  |
| Patients with DJ stent at discharge | 37 (21·9%) | 57 (34.5%) | 0·009 |
| Median (IQR) duration | 28 (21–28) days | 28 (21–28) days | 0·95 |
| **Percutaneous nephrostomy** |  |  |  |
| Patients with PCN | 12 (7·1%) | 32 (19.4%) | 0·001 |
| Duration | 1–2 days | 1–2 days | - |
| PCN present at discharge | 0 | 0 | - |

**Table S5. Subgroup analyses of 30-day symptomatic urinary tract infection by age, surgical approach, procedure type, comorbidity and preoperative urinary drainage. (N=334)**

| **Subgroup** | **Single-dose (n=169)** | | **Extended (n=165)** | | **p-value** |
| --- | --- | --- | --- | --- | --- |
|  | **n** | **UTI (%)** | **n** | **UTI (%)** |  |
| **Overall** | 169 | 12 (7·1%) | 165 | 9 (5·5%) | 0·536 |
| **Age band** |  |  |  |  |  |
| ≤10 years | 2 | 0 (0%) | 2 | 0 (0%) | - |
| 11–69 years | 150 | 9 (6·0%) | 146 | 9 (6·2%) | 0·95 |
| ≥70 years | 17 | 3 (17.6%) | 17 | 0 (0%) | 0·23 |
| **Surgical approach** |  |  |  |  |  |
| Endoscopic | 109 | 8 (7·3%) | 116 | 6 (5·2%) | 0·50 |
| Minimally invasive | 47 | 3 (6·4%) | 36 | 2 (5·6%) | 0·87 |
| Open | 13 | 1 (7·7%) | 13 | 1 (7·7%) | 1·00 |
| **Procedure type** |  |  |  |  |  |
| Upper-tract stone endoscopy (PCNL/RIRS/URSL) | 37 | 6 (16·2%) | 57 | 6 (10·5%) | 0·42 |
| Lower-tract endoscopy (TURBT/TURP/others) | 77 | 2 (2·6%) | 52 | 0 (0%) | 0·52 |
| MIS (laparoscopic/robotic) | 47 | 3 (6·4%) | 36 | 2 (5·6%) | 0·87 |
| Open surgery | 13 | 1 (7·7%) | 13 | 1 (7·7%) | 1·00 |
| **Comorbidity** |  |  |  |  |  |
| Diabetes mellitus | 17 | 1 (5·9%) | 20 | 1 (5·0%) | 1·00 |
| Hypertension | 27 | 2 (7·4%) | 30 | 2 (6·7%) | 1·00 |
| Hypothyroidism | 3 | 1 (33·3%) | 2 | 0 (0%) | 1·00 |
| No comorbidity | 128 | 9 (7·0%) | 117 | 6 (5·1%) | 0·53 |
| **Preoperative urinary drainage** |  |  |  |  |  |
| Double-J ureteric stent | 7 | 2 (28·6%) | 13 | 2 (15·4%) | 0·59 |
| Per-urethral catheter | 21 | 1 (4·8%) | 8 | 0 (0%) | 1·00 |
| Percutaneous nephrostomy | 1 | 0 (0%) | 2 | 0 (0%) | - |
| None | 140 | 9 (6·4%) | 142 | 7 (4·9%) | 0·70 |
