# Supplementary material for: Single-dose/day versus extended antibiotic prophylaxis for elective urological surgery: a prospective randomized controlled trial
Source: eClinicalMedicine. 2026 Oct 29;101:104303. doi: 10.1016/j.eclinm.2026.104303 (PMC13742308; doi:10.1016/j.eclinm.2026.104303)
Supplement: Supplementary file 2 [file mmc2.docx]

**SUBMISSION OF PROTOCOL INVOLVING RESEARCH IN HUMAN SUBJECTS FOR CLEARANCE BY INSTITUTE ETHICS COMMITTEE OF AIIMS**

| 1. Full Title of Study: | Outcome of guideline directed pre-operative prophylactic single dose/day vs extended prophylactic antibotics for urological surgeries: A Randomized controlled study. |  |
| --- | --- | --- |
| 2.1 Name & signatures  of the candidate  2.2 Department  2.3 Degree/course  2.4 Batch of admission to course  2.5 Month & year of submission of thesis | 2.1 Dr Rajath S Shetty.  Signatures________________  2.2 DEPARTMENT OF UROLOGY, AIIMS, NEW DELHI  2.3 MCh (UROLOGY)   2.4 July_2023___ (year)   2.5 January _2026___(year) |  |
| 3. Name of Faculty & Department  (Guide/Co-guide)  3.1 | Guide:  Dr. Rishi Nayyar  Additional Professor  Department of Urology,  AIIMS, New Delhi  Co-Guides:  Dr Sarita Mohapatra  Professor  Department of Microbiology  AIIMS, New Delhi  Dr. Brusabhanu Nayak  Additional Professor  Department of Urology  AIIMS, New Delhi  Dr. Siddharth Jain  Additional Professor  Department of Urology  AIIMS, New Delhi  Dr. Manoj Kumar  Additional Professor  Department of Urology  AIIMS, New Delhi  Dr Sridhar P  Assistant Professor  Department of Urology  AIIMS, New Delhi  Dr Prashant Singh  Assistant Professor  Department of Urology  AIIMS, New Delhi  Dr Amlesh Seth  Professor and Head  Department of Urology  AIIMS, New Delhi |  |

| 4. Objectives of the study | **Primary objective**   - 30-day rate of clinical UTI after urological procedures with single dose/day preoperative antibiotic against extended antibiotic therapy   **Secondary objectives**   - 1-week rate of clinical UTI - 1-week and 30-day bacteriuria rates - 1-week and 30-day asymptomatic bacteriuria rates - 1-week and 30-day septicemia rates - 1-week and 30-day ICU admission rates - length of hospital stay - 1-week and 30-day re-admission rates - Post-op antibiogram resistance patterns - Need for higher antibiotics/change of antibiotics - Antibiotic related side effects or complications |
| --- | --- |
| 5. Why this study is required?  Please provide brief justification. | Post-procedural genito-urinary tract infection (UTI) is a frequent occurrence in the clinical setting with associated morbidity, cost of treatment and possible long term sequelae. Most urological procedures are either Class 1 or 2 procedures with recommended single-shot antibiotic prophylaxis for most. Urological surgeries differ from other surgeries in that (a) Flora that causes surgical site infections is markedly different from cutaneous flora, with more pre-disposition of gram-negative enterobacteriaceae (b) Use of continuous high pressure irrigation in endo-urological surgeries to distend the endoscopic surgical field, resulting in pyelovenous and pyelolymphatic backflow predisposing to development of bacteremia and endotoxiemia, and (c) Frequent use of foreign bodies like stents, catheter or nephrostomy before and after the surgery. As a result, the general recommendations for surgical site infections are not directly applicable to endourological surgery [1]. Even though, most guidelines recommend only a single dose/day of antibiotic prophylaxis for most endourological cases, the use of antibiotics in the peri-operative period is extremely diverse with wide differences between different regions, different hospitals, and even different consultants in same surgical unit. In real-world practice, peri-operative antibiotics use is heavily driven by apprehension of gram-negative bacteremia, endotoxemia and sepsis. Such use stands against the larger community goal of minimizing use of antibiotics wherever feasible. Therefore, there is huge need to reduce this clinical gap between guidelines and actual clinical practice(1). This study seeks to find out if there is any difference between the guideline directed preoperative prophylactic single dose/day vs extended prophylactic antibiotic course for urological surgeries in a prospective randomized manner. |
| 6. Methodology | 6.1. Number of Patients: 334  6.2. Inclusion criteria: Provided in detailed version  6.3. Exclusion criteria: Provided in detailed version  6.4. Control(s) - Single dose/day antibiotic group  6.5. Study design- Randomized Controlled Study  6.6. Dosages of drug- Provided in detailed version  6.7. Duration of treatment – Provided in detailed version  6.8. Investigation specifically related to Protocols: Urine culture at 1 week and 30 week  6.9 Permission to use copyrighted Questionnaire/proforma– Not applicable  6.9. Others: – Not applicable |
| 7. a) Safety measures for proposed  interventions  b) Results of relevant laboratory tests  c) Result of studies in human | a) No special intervention is being done on patients in this study. The group receiving guideline directed single dose/day antibiotics is not at increased risk as per available literature evidence. Nonetheless, the entire cohort shall remain under strict surveillance for entire duration of study as part of protocol. |
| 8. Plans to withdraw standard therapy in research | 🞏 Yes 🗹 No  Remarks: no alternate therapy is instituted to any subject as part of this study. |
| 9. Plan for provision of coverage for  medical risk | No additional/ alternate therapy is instituted to any subject as part of this study. Any infections/sepsis shall be manged as per standard protocols in the urology ward. |
| 10. How you will maintain  Confidentiality of subject? | The information obtained will be kept confidential and only competent authorities like the members of the study team only will have access to the patient records. |
| 11. Costs Involved (Appx. in Rs.) | None |
| 12. Participant Information Sheet  (mark √ if yes) | 🗹 Attached English version  🗹 Attached Hindi version  🗹 *Certified that Hindi version is a true translation of English version* |
| 13. Participant Informed Consent Form (mark √ if yes) | 🗹 Attached English version  🗹 Attached Hindi version  🗹 *Certified that Hindi version is a true translation of English version* |
| 14. Whether any work on this Protocol has started or not? | X (mark √ if yes, X if no)  (Please enclose a separate certificate to this effect). |
| 15.Attached documents  (If any) | 15.1 Covering letter, through proper channel.  15.2 Copy of the detailed protocol is mandatory  15.3 Undertaking that the study shall be done in accordance  with ICMR and GCP guidelines  15.4 In case of multicentric study, IEC clearance of other centres must be provided  15.5 Definite undertaking as to who will bear the expenditure of injury related to the Protocol  15.6 In case an insurance cover is intended, Insurance certificate must be provided (as per ICMR guidelines)  15.7 : Permission as mentioned in 6.9  15.8: Certificate/undertaking as mentioned in 16  15.9 In case of Clinical trials, proof of registration of Clinical trial with ICMR needs to be submitted.  15.10 Others: |

**INTRODUCTION**

Urological procedures have undergone significant advancements, incorporating minimally invasive techniques for diagnosing and treating urological conditions(1). Theoretically endourological procedures, minimally invasive procedures contribute to reduced infection risks compared to traditional open surgeries.

Meticulous perioperative management, especially in the domain of antibiotic use, remains imperative to enhance the outcomes of urological interventions. Antimicrobial prophylaxis (AP) is a cornerstone in mitigating the incidence of surgical site infections (SSI) and other periprocedural infections(2). The primary objective of AP is to reduce SSI while simultaneously curbing antibiotic overuse to counter the rise of multidrug-resistant (MDR) organisms(2). While procedures deemed suitable for without AP can benefit from stringent sterile techniques and best surgical practices, there exists a delicate balance, acknowledging the potential development of MDR organisms and the overall impact on recovery(3).

Despite compelling evidence supporting the efficacy of perioperative antibiotics in diminishing the incidence of postoperative infectious complications in urologic procedures(4), specific guidelines for prophylactic antibiotic regimens, especially in the context of urological procedures remain uncertain (4). This scarcity prompts a critical examination of existing evidence to optimize antibiotic protocols.

Antimicrobial resistance, notably pronounced among urogenital tract pathogens, emphasizes the need for judicious antibiotic use in urological procedures(5). Guidelines must carefully consider the application of AP, striving to minimize its use without compromising postoperative outcomes for individual patients.

Surgical site infections, including urinary tract infections (UTI), impose significant morbidity in urological surgery. The relevance of antibiotic prophylaxis is confined to clean and clean-contaminated operations and is contingent upon the absence of bacterial growth in the urine(6). However, the lack of consistent classification and definitions, coupled with a paucity of well-powered randomized control trials, necessitates a re-evaluation of recommendations for antibiotic prophylaxis in urologic surgery. Current literature suggests a consensus on the need for prophylaxis and antibiotic therapy, with fluoroquinolones demonstrating efficacy in post-operative infection control after percutaneous and ureteroscopic stone removal(3).

Post-procedural genito-urinary tract infection (UTI) is a frequent occurrence in the clinical setting with associated morbidity, cost of treatment, and possible long-term sequelae1. Most urological procedures, categorized as Class 1 or 2, involve recommended single-shot antibiotic prophylaxis. Urological surgeries differ from other surgeries due to distinct factors such as the markedly different flora causing surgical site infections, the use of continuous high-pressure irrigation in endo-urological surgeries, and the frequent use of foreign bodies like stents, catheters, or nephrostomy tubes(1).

Despite most guidelines recommending only a single dose/day of antibiotic prophylaxis for most urological cases, real-world practice shows diverse peri-operative antibiotic use driven by concerns of gram-negative bacteremia, endotoxemia, and sepsis(1). This diversity stands against the larger community goal of minimizing antibiotic use wherever feasible(1).

Hence, recognizing the need to bridge the clinical gap between guidelines and actual practice(1), a study is being conducted to find out if there is any difference between the guideline directed PREOPERATIVE prophylactic SINGLE SHOT ANTIBIOTIC WITH extended prophylactic ANTIBIOTIC course for urological surgeries in a prospective manner

**Review of literature**

Antimicrobial prophylaxis (AP) is a cornerstone in mitigating the incidence of surgical site infections (SSI) and other periprocedural infections(2). The primary objective of AP is to reduce SSI while simultaneously curbing antibiotic overuse to counter the rise of multidrug-resistant (MDR) organisms(2). While procedures deemed suitable for without AP can benefit from stringent sterile techniques and best surgical practices, there exists a delicate balance, acknowledging the potential development of MDR organisms and the overall impact on recovery(3)

Despite compelling evidence supporting the efficacy of perioperative antibiotics in diminishing the incidence of postoperative infectious complications in urologic procedures(4), specific guidelines for prophylactic antibiotic regimens, especially in the context of urological procedures remain uncertain (4).

Antimicrobial resistance, notably pronounced among urogenital tract pathogens, emphasizes the need for judicious antibiotic use in urological procedures This has been explained by an overuse and a frequently extended use of antibiotic agents partly for AP in standard urological procedures (5). Guidelines must carefully consider the application of AP, striving to minimize its use without compromising postoperative outcomes for individual patients.

Surgical site infections, including urinary tract infections (UTI), impose significant morbidity in urological surgery. Despite most guidelines recommending only a single dose/day of antibiotic prophylaxis for most urological cases, real-world practice shows diverse peri-operative antibiotic use driven by concerns of gram-negative bacteremia, endotoxemia, and sepsis(1). Urological surgeries differ from other surgeries due to distinct factors such as the markedly different flora causing surgical site infections, the use of continuous high-pressure irrigation in endo-urological surgeries, and the frequent use of foreign bodies like stents, catheters, or nephrostomy tubes(1).

The claim of single shot antibiotics is backed up numerous studies, such as research conducted by Deborah et al, have indicated that in Class I/clean procedures (i.e., uninfected incisions with no inflammation and/or without entrance into the gastrointestinal or GU tracts), single-dose antibiotic prophylaxis (AP) coverage for usual skin flora may not be necessary. Class I/clean outpatient urologic procedures, such as minimally invasive surgery for renal and adrenal tumors, arteriovenous fistula, and graft creation, have not demonstrated a significant benefit of antibiotic prophylaxis, as outlined in some Class II/clean-contaminated procedures like ureteroscopy (2). Supporting this, a retrospective review conducted by Daniel J. Greene on all adult patients undergoing treatment for stone disease by ureteroscopy/renoscopy with a negative preoperative urine showed, within 30 days after surgery, no significant difference in UTI rates between patients who received and did not receive home-going antibiotics (7). The prophylactic antibiotics administration is more confusing when additional factors when foreign bodies like stents, catheters, or nephrostomy tubes, a study conducted by Madhavan et al showed prophylactic antibiotics have no role in altering bacterial growth on temporary indwelling DJ stent, stent related symptoms or febrile UTIs furthermore it was found that cranberry extract may reduce the colonization of urinary tract, but its clinical significance needs further evaluation(7).

On the contrary, the same is not seen in patients undergoing radical cystectomy with an indwelling stent, prophylactic postoperative antibiotics administered within 30 days following the procedure are associated with a significant decrease in urinary tract infections and a lower rate of readmission from urosepsis (9).

Despite most guidelines recommending only a single dose/day of antibiotic prophylaxis for most urological cases, real-world practice shows diverse peri-operative antibiotic use driven by concerns of gram-negative bacteremia, endotoxemia, and sepsis(1)

Hence, recognizing the need to bridge the clinical gap between guidelines and actual practice(1), a study is being conducted to find out if there is any difference between the guideline directed PREOPERATIVE prophylactic SINGLE SHOT ANTIBIOTIC WITH extended prophylactic ANTIBIOTIC course for urological surgeries in a prospective manner.

**RESEARCH QUESTION**

Is preoperative guideline directed single dose/day antibiotic as effective as extended antibiotic therapy in urological cases to prevent clinical UTI?

**AIMS AND OBJECTIVES**

**AIM**:

To compare the outcome of preoperative prophylactic single dose/day antibiotic with extended prophylactic antibiotics for urological surgeries.

**OBJECTIVES**

**Primary objective**

- 30-day rate of clinical UTI after urological procedures with single dose/day preoperative antibiotic against extended antibiotic therapy

**Secondary objectives**

- 1-week rate of clinical UTI
- 1-week and 30-day bacteriuria rates
- 1-week and 30-day asymptomatic bacteriuria rates
- 1-week and 30-day septicemia rates

**MATERIALS AND METHODS**

This study will be a comparative cohort study. This study will be carried out in the Department of Urology, at the All India Institute of Medical Sciences, New Delhi.

All patients meeting inclusion criteria and giving informed written consent will be included in the study.

**STUDY DESIGN** : Randomised controlled study

**SETTING** : Department of Urology, AIIMS, New Delhi and Microbiology

**i) Inclusion criteria**

a) Patients undergoing elective urological surgeries under general anesthesia.

b) Age ≥5 to ≤ 75 years.

**ii) Exclusion criteria**

a. Patient refusing to consent to study or is unwilling to participate.
b. Cases with opening of Gastro-intestinal tract
c. Reconstructive urological surgeries
d. Artificial implant placement
e. Class 3 or 4 procedure as per WHO criteria(8)
f. Bilateral surgery
g. Pre-operative known focus of active infection
h. immunocompromised status
i. Charlson co-morbidity index of 5 and above(9)
j. patients at risk of infective -endocarditis
k. blood loss > 1 litre
l. pregnancy
m. Any complication during the surgery requiring use of any means other than scheduled procedure,
n. Neuro-urological disease necessitating CIC/indwelling catheter/SPC

**SAMPLE SIZE**: **334**

In the study titled "A Single Dose of Intraoperative Antibiotics Is Sufficient to Prevent Urinary Tract Infection During Ureteroscopy," postoperative urinary tract infection (UTI) rates of 4.7% and 15.3% were reported for single-shot antibiotics and extended-spectrum antibiotics, respectively. The sample size required for this Thesis was determined to be 334, considering a 5% level of significance and 90% power. The calculations were performed using G Power 3.1.9.4 software.

**METHODOLOGY**

1) **Study Enrollment and Consent:** Patients meeting the predetermined inclusion criteria and providing informed consent as detailed in ANNEXURE II and III will be actively recruited for participation in this prospective observational study.
2) **Data Collection - Performa [ANNEXURE I]:** Utilizing a meticulously designed Performa (refer to ANNEXURE I), a systematic collection of data will commence. This will encompass a thorough clinical evaluation, including demographic information (age, gender), relevant medical history, and a detailed examination of symptoms, along with their respective durations.
3) **Basic Investigations:** Subsequent to obtaining explicit consent, participants will undergo a series of basic investigations in accordance with institutional guidelines. These include a comprehensive hemogram, renal function test, liver function tests, chest X-ray, and Urine culture.
4) **Group Stratification and Randomization:** Following initial assessments, enrolled patients will be randomly assigned to either Group 1 or Group 2 using a randomization scheme. Randomization will be done using Block randomization technique to allow for different surgery types (TURP/TURBT/PCNL/URS or RIRS/Nephrectomy/RARP/Adrenalectomy/Others). This rigorous randomization process ensures an unbiased distribution of participants, helping to minimize potential confounding variables and enhance the internal validity of the study. Once randomized

**Group 1 :** Will receive perioperative third-generation cephalosporin (or Culture directed antibiotic), followed by a continuous postoperative regimen of oral/injectable cephalosporin (or Culture directed antibiotic) for 12 doses or more as decided by the surgeon.

**Group 2 :** Will receive perioperative third-generation cephalosporin (or Culture directed antibiotic) as a single dose immediately before the surgical procedure, followed by post-operative antibiotic for maximum allowable duration of 1 Day, calculated from start time of surgery

5) **Post-operative Surveillance:** Vigilant monitoring during the post-operative phase will focus on the emergence of symptoms indicative of Urinary Tract Infection (UTI), including fever, urgency, frequency, and dysuria. In the event of such symptoms, a repeat urinalysis and urine culture will be promptly conducted. Scheduled follow-up urine culture will be organized at 1 week and 30 days to assess for bacteriuria and side effects of antibiotics...
6) **Data Analysis:** Collected data will be subjected to rigorous analysis, employing statistical methods and techniques to draw meaningful conclusions regarding the impact of the administered interventions. This analytical phase aims to contribute valuable insights into perioperative management strategies.
7) **Ethical Considerations:** The study will be conducted in compliance with ethical principles, ensuring patient confidentiality, privacy, and adherence to all relevant ethical guidelines and regulatory requirements.

**STATISTICAL ANALYSIS**

Data will be summarized and analysed using statistical package for social sciences (SPSS, version 24.0) or STATA (version 14) software. Data will be expressed as mean ± standard deviation (SD) or number and percentage for qualitative and quantitative variables as appropriate. The confidence intervals (CI) will be produced with the Wilson Score method. The receiver operating characteristic (ROC) curve and the Fleiss’ kappa coefficient will be used to describe the concordance between the surgeons’ prediction and the result of pathology. The sensitivity, the specificity, the positive predictive value (PPV), and the negative predictive value (NPV) will be calculated using a standard 2 x 2 table comparing the relative ratios of true/ false positives and negatives.

A value of p less than 0.05 will be considered to represent statistical significance of the study.

**BIBLIOGRAPHY**

1.	Clinical Practice Guidelines for Antimicrobial Prophylaxis in Surgery. https://www.ashp.org/surgical-guidelines

2.	Lightner DJ, Wymer K, Sanchez J, Kavoussi L. Best Practice Statement on Urologic Procedures and Antimicrobial Prophylaxis. J Urol. 2020 Feb;203(2):351–6.

3.	Zanetti G, Paparella S, Trinchieri A, Prezioso D, Rocco F, Naber KG. Infections and urolithiasis: current clinical evidence in prophylaxis and antibiotic therapy. Arch Ital Urol Androl. 2008 Mar;80(1):5–12.

4.	Ramaswamy K, Shah O. Antibiotic prophylaxis after uncomplicated ureteroscopic stone treatment: is there a difference? J Endourol. 2012 Feb;26(2):122–5.

5.	Bausch K, Aghlmandi S, Sutter SU, Stamm LM, Ewald H, Appenzeller-Herzog C, et al. Antibiotic prophylaxis in transurethral resection of bladder tumours: study protocol for a systematic review and meta-analysis. Syst Rev. 2020 Dec;9(1):1–6.

6.	Grabe M. Antibiotic prophylaxis in urological surgery, a European viewpoint. Int J Antimicrob Agents. 2011 Dec;38 Suppl:58–63.

7.	Madhavan K, Rustagi S, Jena R, Singh UP, Ansari MS, Srivastava A, et al. A prospective randomized study to define the role of low dose continuous prophylactic antibiotics and anti-adherence agents in altering the microbial colonization related to indwelling double‐J stents. Asian J Urol. 2021 Jul;8(3):269–74.

8.	Herman TF, Bordoni B. Wound Classification. In: StatPearls [Internet]. Treasure Island (FL): StatPearls Publishing; 2023 [cited 2023 Dec 17]. Available from: http://www.ncbi.nlm.nih.gov/books/NBK554456/

9.	Huang Y qun, Gou R, Diao Y shu, Yin Q hua, Fan W xing, Liang Y ping, et al. Charlson comorbidity index helps predict the risk of mortality for patients with type 2 diabetic nephropathy. J Zhejiang Univ Sci B. 2014 Jan;15(1):58–66.

**ANNEXURE I: PATIENT INFORMATION PROFORMA**

Case no.
Name	DOA	Ht

Age/sex	DOO	Wt

CR/UHID no:	DOD	BMI
Address
Occupation	Marital status
Ph no
Pre op diagnosis:

- Urine culture

-Urine Routine

**OPERATIVE DETAILS:**

Operating Surgeon:

- Date of surgery:

-Intraoperative Details (Pus discharge, residual stones)

1. Name
2. Age (completed years)
3. Gender
   a. Male
   b. Female
4. Residential address
5. Phone number (with STD code/mobile)
6. Co-morbid illness / predisposing factors
   a. Diabetes mellitus
   b. Hypothyroid
   c. CKD
   d. Hydro-ureteronephrosis
   e. Previous history of recurrent or peri-operative UTI/sepsis*(even non-urological surgeries)*
   f. Repeat procedure *(within 5 days of first procedure)*
A. **Pre-operative standard bacterial urine culture***
7. Urine culture *(within one week of surgery)*
   a. Not-available
   b. Sterile
   c. Mixed *(multiple bacterial growths)*
   d. Bacterial insignificant count (<10^5^cfu/ml)
      i. Name the bacteria
   e. Bacterial significant count (>10^5^cfu/ml)
      i. Name the bacteria
B. **Procedure type***
8. Surgery done
C. **Procedure Details***
9. If Cystoscopy

*(Inclusion:* *Check Cystoscopy, Diagnostic Cystoscopy, Hydrodistension; Exclusion: Additional OIU, Cystolithotripsy, Post-op catheterization, Stent placement or removal)*

a. Indication(Check Cystoscopy / Diagnostic Cystoscopy / Hydrodistension)
b. History *(within 3 months)* of
   i. Instrumentation
      a) Yes
      b) No
   ii. Catheter free trial
       a) Yes
       b) No
   iii. Previous history of UTI requiring antibiotics
        a) Yes
        b) No

10. If TURP
    a. Prostate size on ultrasound (<30 cc / 31-60 cc / 60-90 cc / >90 cc)
    b. On indwelling catheter
       i. Yes
          a) Duration (<3 days / 4-7 days / >7 days)
       ii. No
           a) Post-void urine volume on ultrasound (<50 cc / 51-150 cc / 151-250 cc/ >251 cc)
    c. History *(within 3 months)*of
       i. Instrumentation
          a) Yes
          b) No
       ii. Catheter free trial
           a) Yes
           b) No
       iii. Previous history of UTI requiring antibiotics
            a) Yes
            b) No
    d. Resection time *(excluding cystoscopy time)*
       a) <45 min
       b) 46-75 min
       c) >75 min
    e. Post-op irrigation time
       a) <6 hours
       b) 6-24 hours
       c) 24-48 hours
       d) >48 hours
    f. Post-op catheterization time
       a) <24 hours
       b) 24-48 hours
       c) 48-72 hours
       d) >72 hours
    g. Post-op TLC (Day 1)
       a) Not done
       b) <10,000 per mm3
       c) 10,001-15000 per mm3
       d) 15,001-20,000 per mm3
       e) >20,000 per mm3
    h. Post-op Clot retention requiring bladder wash
       i. Yes
       ii. No
11. If TURBT
    a. Tumor size on ultrasound (<1 cm / 1.1-3 cm / 3.1-5 cm / >5 cm)
    b. On indwelling catheter
       i. Yes
          a) Duration (<3 days / 4-7 days / >7 days)
       ii. No
           a) Post-void urine volume on ultrasound (<50 cc / 51-150 cc / 151-250 cc/ >251 cc)
    c. History *(within 3 months)* of
       i. Instrumentation
          a) Yes
          b) No
       ii. Catheter free trial
           a) Yes
           b) No
       iii. Previous history of UTI requiring antibiotics
            a) Yes
            b) No
    d. Resection time *(excluding cystoscopy time)*
       a) <45 min
       b) 46-75 min
       c) >75 min
    e. Post-op irrigation time
       a) <6 hours
       b) 6-24 hours
       c) 24-48 hours
       d) >48 hours
    f. Post-op catheterization time
       a) <24 hours
       b) 24-48 hours
       c) 48-72 hours
       d) >72 hours
    g. Post-op TLC (Day 1)
       a) Not done
       b) <10,000 per mm3
       c) 10,001-15000 per mm3
       d) 15,001-20,000 per mm3
       e) >20,000 per mm3
12. If URS
    a. Stone location (Lower ureter / Mid-ureter / Upper ureter / Renal pelvis / Calyx)
    b. Stone size on imaging (<1 cm / 1.1-2 cm / >2 cm)
    c. On urinary diversion
       i. Yes
          a) Type (DJ stent / PCN)
          b) Duration (<3 days / 4-7 days / >7 days)
       ii. No
    d. History *(within 3 months)* of
       i. Instrumentation
          a) Yes
          b) No
       ii. Previous history of UTI requiring antibiotics
           a) Yes
           b) No
    e. Post-op catheterization time
       a) Nil
       b) <6 hours
       c) 6-24 hours
       d) 24-48 hours
       e) >48 hours
    f. Indwelling DJ stent left in situ
       a) Yes
       b) No
    g. Post-op TLC (Day 1)
       a) Not done
       b) <10,000 per mm3
       c) 10,001-15000 per mm3
       d) 15,001-20,000 per mm3
       e) >20,000 per mm3
    h. Residual fragments
       a) Yes
       b) No
13. If PCNL
    a. Number of tracts used (1 / 2 / >2)
    b. Tract size (<15 Fr / 15-21 Fr / 21-25 Fr / >25 Fr)
    c. Largest stone size on imaging (<1 cm / 1.1-2 cm / >2 cm / Partial staghorn / Staghorn)
    d. On urinary diversion
       i. Yes
          a) Type (DJ stent / PCN)
          b) Duration (<3 days / 4-7 days / >7 days)
       ii. No
    e. History *(within 3 months)* of
       i. Instrumentation
          a) Yes
          b) No
       ii. Previous history of UTI requiring antibiotics
           a) Yes
           b) No
    f. Post-op catheterization time
       a) Nil
       b) <6 hours
       c) 6-24 hours
       d) 24-48 hours
       e) >48 hours
    g. Indwelling DJ stent left in situ
       a) Yes
       b) No
    h. Indwelling PCN stent left in situ
       a) Yes
       b) No
    i. Post-op TLC (Day 1)
       a) <10,000 per mm3
       b) 10,001-15000 per mm3
       c) 15,001-20,000 per mm3
       d) >20,000 per mm3
    j. Residual fragments
       a) Yes
       b) No
14. If Nephrectomy
    a. Type of Nephrectomy:
       i. Simple Nephrectomy
       ii. Radical Nephrectomy
       iii. Partial Nephrectomy
    b. Approach:
       i. Open Nephrectomy
       ii. Laparoscopic Nephrectomy
       iii. Robotic Nephrectomy
    c. Indication for Nephrectomy:
       i. Renal mass
       ii. Non-functional Kidney (Stones / UPJO/ ureteric strictute)
       iii. Infective (History of pyonephrosis/ xanthogranulomatous/ emphysematous pyelonephritis / GUTB)
       iv. Other (Specify): _______________
    d. Intraoperative Complications:
       i. Yes

          b) Pus spillage
       ii. No
    e. Type of Urinary Diversion (if applicable):
       i. None
       ii. Ureteral Stent
       iii. PCN _______________
    f. Blood Loss during Surgery (ml):
    g. Blood Transfusion Required
    h. Duration of Surgery:
       i. <2 hours
       ii. 2-4 hours
       iii. >4 hours
    i. Postoperative Complications:
       i. Yes
          a) Hemorrhage
          b) Infection (URI/UTI/SSI/Other)
          c) Urinary Leak
          d) Other (Specify): _______________
       ii. No
    j. Drain Placement:
       i. Yes
       ii. No
    k. Postoperative Catheterization Time:
       i. Nil
       ii. <24 hours
       iii. 24-48 hours
       iv. >48 hours
15. If Adrenalectomy
    a. Type of Adrenalectomy:
       i. Unilateral Adrenalectomy
       ii. Bilateral Adrenalectomy
    b. Approach:
       i. Open Adrenalectomy
       ii. Laparoscopic Adrenalectomy
       iii. Robotic Adrenalectomy
    c. Indication for Adrenalectomy:
       i. Adrenal Mass
       ii. Hormonally Active Tumor
       iii. Suspected Malignancy
       iv. Other (Specify): _______________
    d. Intraoperative Complications:
       i. Yes
          a) Vascular Injury
          b) Other (Specify): ____
       ii. No
    e. Intraoperative Antibiotics Administration:
       i. Yes
       ii. No
    f. Duration of Surgery
       i. <2 hours
       ii. 2-4 hours
       iii. >4 hours
    g. Postoperative Antibiotics Protocol:
       i. Yes:
       ii. No
    h. Postoperative Complications:
       i. yes
          a) Infection
          b) Adrenal Insufficiency
          c) Other (Specify)
       ii. No
    i. Histopathological Examination
       i. Malignant
       ii. Benign
       iii. Not Applicable
    j. Drain Placement:
       i. Yes
       ii. No
    k. Postoperative Catheterization Time
       i. Nil
       ii. <24 hours
       iii. 24-48 hours
       iv. >48 hours
16. RARP
    a. Compliance with Guideline-Directed Prophylaxis:
       i. Yes
       ii. No
    b. Intraoperative Complications:
       i. Yes
          a) Vascular Injury
          b) Pus discharge
          c) Other (Specify): _______________
       ii. No
    c. Console Time:
       i. <2 hours
       ii. 2-4 hours
       iii. >4 hours
    d. Estimated Blood Loss during Surgery:
       i. Minimal
       ii. Blood Transfusion Required
    e. Catheter Placement:
       i. <7 days
       ii. 7-14 days
       iii. >14 days
    f. Postoperative Complications:
       i. Yes
          a) Anastomotic Leak
          b) Urinary Retention
          c) Other (Specify): _______________
       ii. No
    g. Drain Placement:
       i. Yes
       ii. No
    h. Continence Status at Discharge:
       i. Dry
       ii. Mild Incontinence (Pad Use)
       iii. Indwelling Catheter
D. **Antibiotic usage Details***
17. Antibiotic used for treating positive culture before procedure
    a. Yes
       i. Name
       ii. Duration
       iii. Stopped when (> 3days prior / 3 to 1 day prior / continued as peri-operative prophylaxis)
    b. No
18. Antibiotic used for peri-operative prophylaxis
    a. Yes
       i. Name
       ii. Duration (Single dose / 1 day / 2 days / 3 days / >3 days)
    b. No
19. Any additional antibiotic used for peri-operative prophylaxis *(eg. Two or more antibiotics used together for prophylaxis)*
    a. Yes
       i. Name
       ii. Duration (Single dose / 1 day / 2 days / 3 days / >3 days)
    b. No
20. Antibiotic used for post-operative clinical UTI/sepsis *(upto 1 month after surgery)*
    a. Yes
       i. Name
       ii. Duration (1 day / 2-3 days / 3-5 days / >5 days)
    b. No
21. Antibiotic used for post-operative prophylaxis of UTI after discharge
    a. Yes
       i. Name
       ii. Duration (3 days / 3-5 days / 5-10 days / >10 days)
    b. No
22. Reasons for antibiotic use *(if used other than prophylactic peri-operative single dose)* *(Multiple options may be selected, if needed / appropriate)*
    a. Surgeon’s protocol
    b. Institutional protocol
    c. Presence of post-op DJ stent / PCN
    d. Suboptimal surgical asepsis
    e. Suboptimal self-cleanliness by patient/care-giver
    f. Pre-operative risk factor (specify)
    g. Intra-operative risk factor (specify)
    h. Post-operative risk factor
       i. TLC count >10000
       ii. Post-op Fever
       iii. Any other (specify)

**ANNEXURE II: PARTICIPANT INFORMED CONSENT FORM (PICF**)

Protocol / Study number :______________________

Participant identification number for this trial: _______________________
Title of project- “**OUTCOME OF guideline directed PREOPERATIVE prophylactic SINGLE SHOT ANTIBIOTIC WITH extended prophylactic ANTIBIOTICS for urological surgeries: A Randomized controlled study**”.
Name of Principal Investigator: Dr. Rajath S Shetty; Dr. Rishi Nayyar.

Tel.No-8867626477; 9811414607.

The contents of the information sheet dated ______________that was provided have been read carefully by me / explained in detail to me, in a language that I comprehend, and I have fully understood the contents. I confirm that I have had the opportunity to ask questions.

The nature and purpose of the study and its potential risks / benefits and expected duration of the study, and other relevant details of the study have been explained to me in detail. I understand that my participation is voluntary and that I am free to withdraw at any time, without giving any reason, without my medical care or legal right being affected.

I understand that the information collected about me from my participation in this research and sections of any of my medical notes may be looked at by responsible individuals from AIIMS. I give permission for these individuals to have access to my records.

I agree to take part in the above study.

---------------------------------------------	Date:
(Signatures / Left Thumb Impression)	Place:
Name of the Participant: _____________________________________
Son / Daughter / Spouse of: ___________________________________
Complete postal address: _____________________________________
This is to certify that the above consent has been obtained in my presence.
------------------------------	Date:
Signatures of the Principal Investigator	Place:
1)	Witness – 1	2)	Witness – 2
------------------------------	--------------------------------
Signatures	Signatures
Name:	Name:
Address:	Address:

**अनुलग्नक II: प्रतिभागी सूचित सहमति फॉर्म (पीआईसीएफ)**

प्रोटोकॉल / अध्ययन संख्या: ______________________

इस परीक्षण के लिए प्रतिभागी पहचान संख्या: _______________________

परियोजना का शीर्षक- " यूरोलॉजिकल सर्जरी के लिए मार्गनिर्देशित पूर्व-ऑपरेटिव सुरक्षात्मक एकल शॉट एंटीबायोटिक का परिणाम और विस्तारित सुरक्षात्मक एंटीबायोटिक्स का तुलना करने के लिए: एक रैंडमाइज्ड कंट्रोल्ड अध्ययन।"

प्रिंसिपल इनवेस्टिगेटर का नाम: डॉ रजत एस शेट्टी; डॉ ऋषि नैय्यर।

दूरभाष (ओं) 8867626477; 9811414607.

दिनांक ______________ की सूचना पत्रक की सामग्री प्रदान की गई थी, जो मुझे समझने वाली भाषा में, मेरे द्वारा विस्तार से समझाया गया है / समझाया गया है, और मैंने सामग्री को पूरी तरह से समझ लिया है। मैं पुष्टि करता हूं कि मुझे प्रश्न पूछने का अवसर मिला है।

अध्ययन की प्रकृति और उद्देश्य और इसके संभावित जोखिम / लाभ और अध्ययन की अपेक्षित अवधि, और अध्ययन के अन्य प्रासंगिक विवरणों को विस्तार से समझाया गया है। मैं समझता हूं कि मेरी भागीदारी स्वैच्छिक है और मैं किसी भी समय बिना किसी कारण के, मेरी चिकित्सा देखभाल या कानूनी अधिकार प्रभावित होने के बिना वापस लेने के लिए स्वतंत्र हूं।

मैं समझता हूं कि इस शोध में मेरी भागीदारी से मेरे बारे में एकत्र की गई जानकारी और मेरे किसी भी मेडिकल नोट के अनुभाग एम्स से जिम्मेदार व्यक्तियों द्वारा देखे जा सकते हैं। मैं इन व्यक्तियों के लिए अपने रिकॉर्ड तक पहुंचने की अनुमति देता हूं।

मैं उपरोक्त अध्ययन में भाग लेने के लिए सहमत हूं।

---------------------------------------------	दिनांक:

(हस्ताक्षर / बाएं थंब इंप्रेशन)	जगह:

प्रतिभागी का नाम: _____________________________________

पुत्र / बेटी / पति / पत्नी: ___________________________________

पूरा डाक पता: _____________________________________

यह प्रमाणित करना है कि उपर्युक्त सहमति मेरी उपस्थिति में प्राप्त की गई है।

------------------------------	दिनांक:

प्रिंसिपल इनवेस्टिगेटर के हस्ताक्षर	जगह:

1) साक्षी - 1	2) साक्षी - 2

------------------------------	--------------------------

हस्ताक्षर	हस्ताक्षर

नाम: नाम:

पता: पता

**ANNEXURE III : PARTICIPANT INFORMATION SHEET (PIS)**

i) Title of the Study/Project:

OUTCOME OF guideline directed PREOPERATIVE prophylactic SINGLE SHOT ANTIBIOTIC WITH extended prophylactic ANTIBIOTICS for urological surgeries: A Randomized controlled study**.**

ii) **Aims and methods of the research**.

We would like to invite you to participate in a study conducted by the Department of Urology, AIIMS, Delhi. The aim of this study is. The findings will provide valuable insights into the diagnostic value of Antibiotic regime, leading to potential improvements in patient management strategies.

In this study, you will undergo a preoperative assessment, including a comprehensive history-taking to evaluate their condition prior to the operation. All relevant investigations, such as imaging studies and blood tests, including Urine routine and urine culture. The surgical procedures performed during the operation will adhere to the standard operative protocols practiced in the surgical unit at the time.

**iii)Expected duration of the subject participation**.

During the course of admission for your surgery to a maximum of 30 days if symptoms present.

iv) **The benefits to be expected from the research to the subject or to others**.

This study aims to assess the significance of Perioperative antibiotic coverage to reduce overuse and abuse

v) **Any risk to the subject associated with the study.**

None. There is no deviation from your standard treatment will be done as part of this study.

**vi)** **Maintenance of confidentiality of records.**

Your participation will be kept confidential. Your medical records will be amenable only to personnel involved in the study. The results of this study may be presented in any conference or published in a journal, but your identity will be completely anonymous.

**vii)** **Provision of free treatment for research related injury**.

In this study, no intervention is being done to cause any research related injury .

**viii)** **Compensation of subjects for disability or death resulting from such injury.**

There is no provision of insurance in this study. No compensation will be paid for any disability or death resulting from such injury. No deviation from the standard treatment for your medical condition is being done as part of this study.

**ix)** **Freedom of individual to participate and to withdraw from research at any time without penalty or loss of benefits to which the subject would otherwise be entitled**.

Your participation in this study is optional and voluntary. You are free to withdraw from this research anytime as per your convenience and this will not affect your subsequent medical treatment with the treating physician.

**x)** **Telephone number/contact number of Principal Investigator and Co-investigator at the top of each page**.

The same is provided for your convenience

**xi)** **Self certification should be given that translation to vernacular is accurate.**

It is certified that the translation to vernacular language is accurate.

**xii)** **Statement that there is a possibility of failure of IP to provide intended therapeutic effect**.

No drug used for this study

**xiii)** **Statement that in case of placebo controlled trials, the placebo administered to the subjects shall not have any therapeutic effect**

**xiv) How will your groups be divided**

**Group 1:** Will be administered perioperative third-generation cephalosporin, followed by continuous oral/injectable cephalosporin for one week postoperatively.

**Group 2:** Will be administered perioperative third-generation cephalosporin as a single shot before the surgical procedure**.**

**xv)**	**Follow up :** Patient will be followed up for a period of 30 days for symptoms of UTI, such as fever, burning micturition. If any symptoms arise appropriate tests will be done treatment will be provided.

**xvi)** No placebo used.

Principal Investigators:

**Dr. RAJATH S SHETTY	Prof. Dr. Rishi Nayyar**

Senior Resident	Additional Professor

Department of Urology	Department of Urology

AIIMS, New Delhi	AIIMS, New Delhi

Mob:8867626477	Mob: 9811414607

**अनुलग्नक III: साझेदार सूचना पत्र (पीआईएस)**

i) अध्ययन / परियोजना का शीर्षक:

यूरोलॉजिकल सर्जरी के लिए मार्गनिर्देशित पूर्व-ऑपरेटिव सुरक्षात्मक एकल शॉट एंटीबायोटिक का परिणाम और विस्तारित सुरक्षात्मक एंटीबायोटिक्स का तुलना करने के लिए: एक रैंडमाइज्ड कंट्रोल्ड अध्ययन।

ii) अनुसंधान के लक्ष्य और तरीके।

हम आपको यूरोलॉजी विभाग, एम्स, दिल्ली द्वारा आयोजित एक अध्ययन में भाग लेने के लिए आमंत्रित करना चाहते हैं। इसका उद्देश्य यूरोलॉजिकल सर्जरी के लिए विस्तारित रोगनिरोधी एंटीबायोटिक्स के साथ प्रीऑपरेटिव रोगनिरोधी सिंगल शॉट एंटीबायोटिक निर्देशित दिशानिर्देश का परिणाम जानना हैं। निष्कर्ष एंटीबायोटिक शासन के नैदानिक मूल्य की समझ को बढ़ाने में योगदान देंगे, जिससे संभावित रूप से रोगी प्रबंधन रणनीतियों में सुधार होगा।

इस अध्ययन के लिए ऑपरेशन से पहले आपके लक्षणों का विस्तृत इतिहास लेकर आपकी स्थिति के संबंध में मूल्यांकन किया जाएगा। सभी प्रासंगिक जांच, जैसे इमेजिंग अध्ययन और रक्त परीक्षण, जिसमें इंट्राऑपरेटिव टीयूआरबीटी वीडियो भी शामिल है, रिकॉर्ड किया जाएगा। आपका ऑपरेशन आपकी सर्जरी के समय प्रचलित सर्जिकल यूनिट में प्रचलित मानक ऑपरेटिव प्रक्रियाओं के अनुसार होगा, जिसे आगे की समीक्षा के लिए रिकॉर्ड किया जाएगा। डिस्चार्ज होने के बाद, आपकी अंतिम हिसपैथोलॉजी का मूल्यांकन किया जाएगा।

iii) विषय भागीदारी की अपेक्षित अवधि।

आपकी सर्जरी के लिए प्रवेश के दौरान से लेकर अधिकतम 30 दिन यदि लक्षण मौजूद हों तो।

iv)	अनुसंधान से विषय या दूसरों के लिए होने वाले लाभों की अपेक्षा की जा सकती है।

इस अध्ययन का उद्देश्य अति प्रयोग और दुरुपयोग को कम करने के लिए पेरीऑपरेटिव एंटीबायोटिक कवरेज के महत्व का आकलन करना है।

v) अध्ययन से जुड़े विषय के लिए कोई जोखिम।

कोई नहीं। इस अध्ययन के भाग के रूप में आपके मानक उपचार से कोई विचलन नहीं किया जाएगा।

vi) अभिलेखों की गोपनीयता का रख-रखाव।

आपकी भागीदारी गोपनीय रखी जाएगी। आपके मेडिकल रिकॉर्ड केवल अध्ययन में शामिल कर्मियों के लिए उपयुक्त होंगे। इस अध्ययन के परिणाम किसी भी सम्मेलन में प्रस्तुत किए जा सकते हैं या जर्नल में प्रकाशित हो सकते हैं, लेकिन आपकी पहचान पूरी तरह से अज्ञात होगी।

vii) अनुसंधान से संबंधित चोट के लिए नि: शुल्क उपचार की व्यवस्था।

इस अध्ययन में, किसी भी शोध-संबंधी चोट के कारण इस अध्ययन के हिस्से के रूप में कोई हस्तक्षेप नहीं किया जा रहा है।

viii) विकलांगता या मृत्यु के लिए विषयों की मुआवजा जिसके परिणामस्वरूप चोट।

इस अध्ययन में बीमा का कोई प्रावधान नहीं है। ऐसी चोट से होने वाली किसी भी विकलांगता या मृत्यु के लिए कोई मुआवजा नहीं दिया जाएगा। इस अध्ययन के हिस्से के रूप में आपकी चिकित्सा स्थिति के लिए मानक उपचार से कोई विचलन नहीं किया जा रहा है।

ix) किसी भी समय जुर्माना या लाभ के नुकसान के बिना व्यक्तिगत रूप से भाग लेने और अनुसंधान से वापस लेने के लिए व्यक्ति की स्वतंत्रता, जिसके विषय में अन्यथा हकदार होगा।

इस अध्ययन में आपकी भागीदारी वैकल्पिक और स्वैच्छिक है। आप वापस लेने के लिए स्वतंत्र हैं

आपकी सुविधा के अनुसार किसी भी समय इस शोध से और इससे आपके प्रभावित नहीं होंगे

उपचार चिकित्सक के साथ बाद में चिकित्सा उपचार।

x) चाय चम्मच पूर्ण में, मात्रा में रक्त नमूना की मात्रा लेने के लिए

उल्लेख किया जाना चाहिए।

नियमित रूप से, इस अध्ययन के हिस्से के रूप में कोई रक्त नमूना नहीं लिया जाएगा। आप नहीं गुजरेंगे

इस अध्ययन के उद्देश्य के लिए कोई अतिरिक्त जांच या उपचार।

xi) प्रिंसिपल इनवेस्टिगेटर और सह के टेलीफोन नंबर / संपर्क संख्या प्रत्येक पृष्ठ के शीर्ष पर जांचकर्ता।

आपकी सुविधा के लिए भी प्रदान किया जाता है

xii) स्व प्रमाणीकरण दिया जाना चाहिए कि स्थानीय भाषा में अनुवाद है सही।

यह प्रमाणित है कि स्थानीय भाषा का अनुवाद सटीक है।

xiii) वक्तव्य है कि इरादे प्रदान करने के लिए आईपी की विफलता की संभावना है उपचारात्मक प्रभाव।

इस अध्ययन के लिए कोई दवा नहीं है

xiv) फॉलो-अप: मरीज़ों को 30 दिनों तक फॉलो- अप किया जाएगा।, जिसमें पेशाब की रास्ते में संक्रमण के इलाज को देखा जाएगाI जैसे कि बुखार, पेशाब करते समय जलन, बार-बार पेशाब आना । अगर कोई लक्षण आते हैं, तो उचित टेस्ट किए जाएंगे और इलाज किया जाएगा।

xv) समूह 1: ऑपरेशन के दौरान दवा दी जाएगी, जिसके बाद एक सप्ताह तक लगातार दवा खाई जाएगी या सुई लगाई जाएगी (विस्तारित स्पेक्ट्रम वाली)।

• समूह 2: सर्जिकल प्रक्रिया से पहले एक ही बार में एक डोज दवा दी जाएगी।

प्रिंसिपल इनवेस्टिगेटर का नाम: डॉ रजत एस शेट्टी; डॉ ऋषि नैय्यर।

दूरभाष (ओं) 8867626477; 9811414607
